# Radiation-specific sexual and reproductive health challenges in adolescent and young adults assigned female at birth: a mixed-methods study of unmet needs post pelvic radiotherapy

**DOI:** 10.1016/j.gore.2026.102051

**Published:** 2026-02-22

**Authors:** Kaviya Devaraja, Anjali Sachdeva, Karina Gandhi, Abha A. Gupta, Jennifer Croke

**Affiliations:** a Institute of Medical Science, University of Toronto, Toronto, Canada; b Adolescent and Young Adult Program, Department of Supportive Care, Princess Margaret Cancer Centre, University of Toronto, Toronto, Canada; c Temerty Faculty of Medicine, University of Toronto, Toronto, Canada; d Department of Psychology, University of Guelph, Guelph, Canada; e Division of Medical Oncology and Hematology, Princess Margaret Cancer Centre, University of Toronto, Toronto, Canada; f Radiation Medicine Program, Princess Margaret Cancer Centre, University Health Network, Toronto, Canada

**Keywords:** SRH, Adolescent and young adult, Sexual dysfunction, Fertility, Pelvic radiotherapy, Cancer, Gynecological malignancies

## Abstract

**Purpose:**

Pelvic radiotherapy (RT) is an effective treatment for pelvic malignancies but often leads to sexual and reproductive health (SRH) challenges, particularly among adolescent and young adult (AYA) patients assigned female at birth (AFAB). Despite known toxicities, no study has systematically explored the SRH experiences, challenges, and unmet care needs of this population post-RT. This study aimed to assess SRH impacts and identify gaps in care among AYA AFAB cancer survivors following pelvic RT.

**Patients and Methods:**

We conducted a simultaneous mixed-methods study at a tertiary cancer center. AYA AFAB patients aged 18–39 who completed pelvic RT between 2018 and 2023 completed a 23-item survey assessing SRH experiences and care satisfaction; a subset participated in in-depth interviews. Quantitative data were analyzed descriptively, and qualitative data were examined using thematic analysis. Triangulation enabled a comprehensive understanding of patient experiences.

**Results:**

Fifty-two participants completed the survey (median age 33), and 15 completed interviews. Most reported sexual dysfunction (90%), intimacy disruption (90%), and fertility-related distress (87%). Three themes emerged: (1) RT-induced sexual dysfunction negatively affected relationships and intimacy, (2) fertility and reproductive disruptions were compounded by inadequate education and support, and (3) chronic pelvic discomfort and body image changes caused emotional and psychosocial distress. Participants emphasized limited provider communication, scarce RT-specific SRH resources, and lack of post-treatment supports.

**Conclusions:**

This first study characterizing RT-specific SRH disruptions among AYA AFAB survivors underscores the urgent need for age-appropriate survivorship care integrating SRH education, counseling, and partner-inclusive supports.

## Highlights

• First mixed-methods study of AYA AFAB survivors post-pelvic radiotherapy.
• 90% reported sexual dysfunction and intimacy disruption after radiotherapy.
• Fertility loss and reproductive distress often lacked tailored education.
• Chronic pelvic pain and body image changes caused emotional distress.
• Identifies need for RT-specific, partner-inclusive SRH survivorship care.

## 1 Introduction

Sexual and reproductive health (SRH) is a critical concern for individuals diagnosed with cancer and one of the most important domains of survivorship for adolescents and young adults (AYAs, aged 15–39 years) (Oveisi et al., 2025, Cherven et al., 2022). AYAs face unique developmental and psychosocial challenges because of their diagnosis and treatments during a formative life stage characterized by exploration of sexual identity, relationships, and future goals, including parenthood (Barnett et al., 2016, Reinman et al., 2021). Among individuals assigned female at birth (AFAB), treatment of pelvic malignancies commonly involves pelvic radiotherapy (RT), while effective, can result in acute and late side-effects, including sexual toxicity, premature ovarian failure, and infertility (Viswanathan et al., 2014). Despite these well-recognized toxicities, the SRH needs of this population remain poorly understood and under-addressed in clinical practice and research (Lehmann et al., 2021).

The World Health Organization (WHO) defines SRH as a state of physical, emotional, mental, and social well-being related to sexuality and reproduction, including the ability to have a safe, satisfying sex life and make informed reproductive decisions (World Health Organization, 2025). For AYA AFAB, pelvic RT threatens these domains through mechanisms including vaginal scarring, stenosis, and ovarian failure (Varyte and Bartkeviciene, 2021, Wo and Viswanathan, 2009); frequently causing sexual dysfunction (e.g., reduced desire, arousal difficulties, and dyspareunia) (Guedes et al., 2022, Menon and Gadiraju, 2025).

These sequelae carry psychological burdens, including distress, diminish self-esteem, and strain on intimate relationships, with fertility concerns closely tied to SRH for AYAs AFAB (Menon and Gadiraju, 2025, Reiser et al., 2024). Pelvic RT-related vaginal atrophy and hormonal disruption impair both sexual function and fertility, intensifying distress regarding identity, body image, and future family-building during this developmental period when such disruptions have disproportionate impacts (Simon et al., 2014, Smith et al., 2024). This highlights the importance of proactive, provider-led SRH and fertility discussions during and after cancer care (Alvers et al., 2020). Although the American Society of Clinical Oncology (ASCO) guidelines encourage provider-initiated discussions on SRH and fertility for all cancer patients (Carter et al., 2017), these conversations are often infrequent, inadequately detailed, and inconsistently offered, particularly for AYAs AFAB receiving pelvic RT (Albers et al., 2020, Darabos et al., 2022), a concerning gap given the irreversible consequences of delayed or absent intervention and the developmental priorities of this group (Barnett et al., 2016).

Despite widespread acknowledgment of these issues, research on SRH experiences of AYA AFAB following pelvic RT resulting is limited. Existing studies have often examined broader populations such as mixed-age cohorts, cancer types, or treatments, in which individuals with pelvic malignancies and/or those treated with pelvic RT represent only a subset rather than the primary focus (Oveisi et al., 2025, Benedict et al., 2025). As a result, findings from this literature are not fully transferable to AYA AFAB who receive pelvic RT specifically. Pelvic RT uniquely impacts structures critical to sexual function (e.g., vaginal tissues, ovaries, pelvic floor) (Bernard et al., 2016), understanding the lived SRH experiences of this population is essential to advancing patient-centered care and developing appropriate guidelines and resources. To date, no study has systematically explored the SRH experiences of AYA AFAB survivors of pelvic RT, nor how their educational and support needs are addressed in oncology practice.

To address this gap, our study is amongst the first to examine the SRH experiences, challenges, and unmet needs of AYAs AFAB who have undergone pelvic RT. Our objectives were: (1) assess SRH challenges experienced by this population following pelvic RT; and (2) understand the education and support received, identify care gaps, and inform development of SRH-focused resources tailored to this group. We conducted our study at a high-volume tertiary cancer center to gather insights that inform clinical care, guide survivorship programming, and shape future research in this long-overlooked area of AYA oncology.

## 2 Methods

### 2.1 Study Population

A simultaneous mixed-methods approach was used to collect and analyze both quantitative (survey) and qualitative (interview) data, enabling triangulation, the integration and cross-validation of findings across data sources, to strengthen validity and allowing qualitative insights to contextualize quantitative trends (Green et al., 2016).

Eligible participants were English-speaking AYA patients AFAB, aged 18–39, completed RT with curative or palliative intent between January 2018 and December 2023 for a primary diagnosis of gynecological cancers (predominantly cervical cancer) gastrointestinal cancers, lymphoma or sarcoma. The majority of our study population consisted of gynecologic oncology patients, and all gynecologic participants had cervical cancer**,** reflecting the core population of interest. RT included external beam radiation to the pelvic organs or brachytherapy. Patients were first identified in the MOSAIQ oncologic database based on receipt of pelvic RT, after which study eligibility criteria were applied. Primary care physician at University Health Network (UHN) were contacted to confirm appropriateness of outreach. Eligible participants were then approached by their clinical team, followed by contact from the study lead (K.D.) for consent. We sought a convenience sample of ∼ 50 AYAs to capture a diverse range of diagnoses and experiences, while ensuring feasibility based on clinic volume and research capacity. Ethics approval was obtained from the UHN Research Ethics Board (22–6012).

### 2.2 Surveys − Recruitment, Data Collection, Analysis

Participants completed a 23-item online survey with three sections: 1) demographics, 2) patient-reported SRH, and 3) SRH care received (Supplemental Table S1).

The patient-reported SRH section assessed the post-treatment experiences, challenges, needs, and quality of life. The SRH care section assessed informational, physical, psychosocial, and resource support received during treatment, along with care adequacy and suggested improvements. No existing validated instrument comprehensively addressed SRH across clinical and psychosocial domains specific to AYA AFAB post-pelvic RT. Therefore, survey items were developed by authors based on literature and current clinical guidelines to ensure they address relevant issues and capture comprehensive data on patient experiences (Dizon et al., 2024, Jensen and Froeding, 2015). Because most participants received multimodality therapy, survey items were explicitly framed around pelvic radiotherapy (PRT) to anchor responses to RT-related experiences, capturing patient-attributed impacts rather than attempting to causally isolate RT effects from surgery or systemic therapy. Quantitative data were analyzed using descriptive statistics to summarize demographics and response trends.

### 2.3 Interviews − Recruitment, Data Collection, Analysis

At the end of the survey, participants were invited to participate in virtual one-on-one interviews, conducted by a member of our study team (K.D.). The interview guide was informed by literature review and expert consultation to comprehensively explore patient SRH experiences and needs (Oveisi et al., 2023) (Supplemental Table S2). To distinguish RT-related effects within the context of multimodality treatment, interview questions and probes were explicitly anchored to participants’ experiences of PRT, and analysis focused on patient-attributed RT-specific impacts while acknowledging overlap with surgery and systemic therapy.

Interviews were conducted and reviewed, thematic saturation was deemed achieved when no new codes or themes emerged in consecutive interviews and existing themes were sufficiently rich and repetitive across participants (Saunders et al., 2018). Saturation was reached after 15 interviews, at which point additional interviews were judged unlikely to yield novel insights. Interviews were audio recorded, transcribed verbatim, and de-identified. Thematic analysis was performed to identify patterns and themes (Braun and Clarke, 2006). Three researchers (K.D., A.S., and K.G.) independently coded transcripts, organizing codes into themes. Discrepancies in coding were resolved through team discussion and consensus, resulting in a shared coding framework to ensure consistency.

Survey and interview findings were integrated during analysis to enable triangulation and nuanced understanding of patient experiences. Interview themes were compared with survey data to confirm, expand, or contextualize patterns across both sources.

## 3 Results

**Fig. 1:**
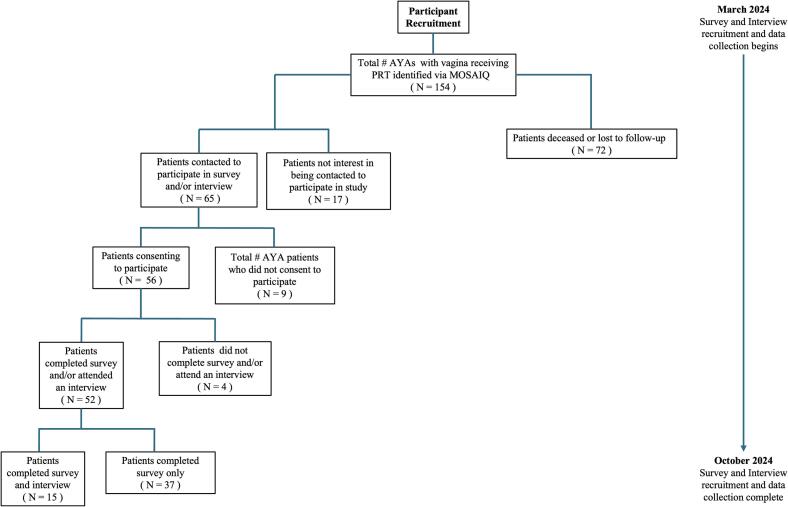
Participant recruitment flow chart.

Between March and October 2024, 52 patients completed the survey, and of these, 15 agreed to participate in one-on-one interviews (Fig. 1).

### 3.1 Demographics

Median age was 33 (18–39), with the majority of patients aged 30–39, and most were gynecologic oncology patients, all of whom were diagnosed with cervical cancer (43/52, 82.7%), reflecting the primary focus population of this study. The response rate for this study was 52/74 (70.3%). While all patients received RT, treatment type varied based on cancer type and stage, with some receiving external beam RT to the pelvis alone (17/52, 32.7%), brachytherapy alone (4/52, 7.7%), or a combination of both (31/52, 59.6%), surgery (47/52, 90.4%), and systematic therapy (44/52, 84.6%). Most participants also underwent surgery (47/52, 90.4%), including total abdominal hysterectomy (19/52, 36.5%), unilateral or bilateral salpingo-oophorectomy (20/52, 38.5%), and fertility-sparing surgery (19/52, 36.5%); some participants underwent more than one surgical procedure. Systemic therapy was received by 44/52 (84.6%). All participants self-identified as female (cis gender), with 79% identifying as heterosexual, and most (32/52, 61.5%) reporting they were currently sexually active. Half were partnered (26/52, 50.0%), and most (34/52, 65.4%) reported their partners also experienced sexual challenges because of the patient’s dysfunction (Table 1). Median time for questionnaire completion from treatment was 26 months (3–82 months).

**Table 1:** Demographics of Study Participants.

**Characteristics**		**Total Patients Survey (n, %)** **N = 52**
**Age at Treatment**	15–19	5 (9.6)
	20–29	6 (3.8)
	30–39	41 (78.8)
**Gender Identity**	Female	52 (100.0)
	Male	0 (0.0)
	Non-binary	0 (0.0)
**Primary Cancer Diagnosis**	Gynecological	43 (82.7)
	Sarcoma	5 (9.6)
	Lymphoma	2 (3.8)
	Gastrointestinal	2 (3.8)
**Radiotherapy**	External Beam Radiotherapy (EBRT)	17 (32.7)
	Brachytherapy (BT)	4 (7.7)
	External Beam Radiotherapy (EBRT) + Brachytherapy (BT)	31 (59.6)
**Treatment**	Radiotherapy	52 (100.0)
	Systematic Therapy	44 (84.6)
	Surgery	47 (90.4)
**Type of Gynecological Surgery**	Total Abdominal Hysterectomy (TAH)	19 (36.5)
	Unilateral/Bilateral Salpingo-oophorectomy (USO/BSO)	20 (38.5)
	Fertility Sparing Surgery	19 (36.5)
**Type of Systematic Therapy**	Chemotherapy	44 (84.6)
	Immunotherapy	2 (3.8)
	Targetted Therapy	3 (5.8)
	Hormonal Therapy	15 (28.8)
**Sexual Orientation**	Heterosexual	41 (78.8)
	Bisexual	4 (7.7)
	Lesbian	3 (5.8)
	Queer	1 (1.9)
	I prefer not to answer	3 (5.8)
**Current Sexual Activity**	Yes	32 (61.5)
	No	15 (28.8)
	I prefer not to answer	5 (9.6)
**Relationship Status**	Partnered	26 (50.0)
	Not partnered	20 (38.5)
	I prefer not to answer	6 (11.5)
**Partner Sexual Challenges Related to Participant’s Sexual Dysfunction**	Yes	34 (65.4)
	No	13 (25.0)
	I prefer not to answer	5 (9.6)
**Education Completed**	Elementary/Middle School	2 (3.8)
	High school diploma	21 (40.4)
	University or college degree	16 (30.8)
	Graduate or professional degree	10 (19.2)
	I prefer not to answer	3 (5.8)
**Employment Status**	Student	12 (23.1)
	Part-time	7 (13.5)
	Full-time	23 (44.2)
	On disability	4 (7.7)
	Not employed	3 (5.8)
	I prefer not to answer	3 (5.8)
**Self-reported Other Health Conditions**	None	27 (51.9)
	Another cancer diagnosis	9 (17.3)
	Mental health (e.g., anxiety)	10 (19.2)
	Drug and Alcohol Abuse	1 (1.9)
	Obesity	2 (3.8)
	Eating disorders	3 (5.8)
**Net Household Income**	Less than $90,000	20 (38.5)
	$91,000-$150,999	14 (26.9)
	Greater than $150,000	8 (15.4)
	I prefer not to answer	10 (19.2)

### 3.2 Findings

The triangulation of survey data and interview content revealed several challenges commonly experienced among AYAs with cancer, such as delays in life-stage milestones like family planning (47/52, 90.4%), disruptions to intimate relationships (47/52, 90.4%), and the emotional toll of managing both treatment and post-treatment concerns (44/52, 80.0%). However, AYAs AFAB receiving RT also endorsed unique challenges specific to treatment including psychosocial, physical, and SRH impacts.

Three themes emerged: 1) managing the impact of pelvic RT-inducted sexual dysfunction on relationships and intimacy, 2) navigating the impact of pelvic RT-specific SRH changes on fertility and family planning, and 3) understanding the emotional and psychosocial toll of chronic pelvic discomfort and post-pelvic RT treatment body changes.

#### 3.2.1 Managing the impact of pelvic RT-Induced sexual dysfunction on relationships and intimacy

Among 52 survey respondents, 90% (n = 47) reported experiencing loss of intimacy due to changes after pelvic RT, 75% (n = 39) experienced deterioration of relationship communication, 73% (n = 38) reported loss of sexual desire linked to pelvic pain and dryness, and 69% (n = 36) reported persistent pain during sex after pelvic RT (Table 2). Most respondents (85%, n = 44) reported that pelvic RT specifically negatively impacted their intimate relationships, both physically and emotionally. Despite these challenges, key rehabilitative interventions were underutilized, 42% (n = 22) had not used vaginal dilators post-radiation, 67% (n = 35) had not accessed marital/relationship counseling, and 73% (n = 38) had not received SRH counseling. Twenty-seven percent (n = 14) received SRH counseling, of these 71% (n = 10) were dissatisfied with the quality of SRH counseling they received. Half (50%, n = 7) felt the counseling did not address their pelvic RT-related concerns, and 64% (n = 9) stated it did not help them manage SRH challenges during or after treatment.

**Table 2:** Survey Responses.

**Question**	**Selection (N %)**				
1. **What changes to sexual health do you experience now?**	**Not at all**	**Rarely**	**Sometimes/ Occasionally**	**Frequently / Often**	**Always**
Vaginal dryness	17 (32.7)	9 (17.3)	9 (17.3)	14 (26.9)	3 (5.8)
Vaginal stenosis	31 (59.6)	8 (15.4)	6 (11.5)	5 (9.6)	2 (3.8)
Vaginal discomfort	13 (25.0)	9 (17.3)	20 (38.5)	10 (19.2)	0 (0.0)
Pain during sex	5 (9.6)	11 (21.2)	12 (23.1)	7 (13.5)	17 (32.7)
Loss of libido or sexual desire	5 (9.6)	9 (17.3)	13 (25.0)	16 (30.8)	9 (17.3)
Loss of enjoyment in sexual activity	7 (13.5)	9 (17.3)	19 (36.5)	5 (9.6)	12 (23.1)
Loss of engagement in sexual activity	5 (9.6)	11 (21.2)	22 (42.3)	5 (9.6)	9 (17.3)
Changes in orgasms or intensity of orgasms	25 (48.1)	7 (13.5)	11 (21.2)	3 (5.8)	6 (11.5)
Anxiety	4 (7.7)	8 (15.4)	20 (38.5)	14 (26.9)	6 (11.5)
Depression	6 (11.5)	14 (26.9)	8 (15.4)	11 (21.2)	13 (25.0)
Emotional distress	8 (15.4)	12 (23.1)	10 (19.2)	7 (13.5)	15 (28.8)
Body image concerns	10 (19.2)	4 (7.7)	10 (19.2)	18 (34.6)	10 (19.2)
Loss of intimacy	5 (9.6)	12 (23.1)	10 (19.2)	14 (26.9)	11 (21.2)
Deterioration of relationship communication	13 (25.0)	25 (48.1)	7 (13.5)	4 (7.7)	3 (5.8)
Changes in self-esteem	8 (15.4)	4 (7.7)	15 (28.8)	20 (38.5)	5 (9.6)
Fatigue	13 (25.0)	6 (11.5)	13 (25.0)	12 (23.1)	8 (15.4)
Fertility issues	7 (13.5)	5 (9.6)	10 (19.2)	4 (7.7)	26 (50.0)
Pelvic pain	14 (26.9)	9 (17.3)	11 (21.2)	11 (21.2)	7 (13.5)
Bowel incontinence	29 (55.8)	2 (3.8)	12 (23.1)	6 (11.5)	3 (5.8)
Bladder incontinence	13 (25.0)	4 (7.7)	15 (28.8)	11 (21.2)	9 (17.3)
Lymphedema	40 (76.9)	3 (5.8)	4 (7.7)	3 (5.8)	2 (7.7)
Diarrhea	28 (53.8)	8 (15.4)	6 (11.5)	10 (19.2)	0 (0.0)
Cramping	31 (59.6)	12 (23.1)	5 (9.6)	0 (0.0)	4 (7.7)
1. **For each statement below, please indicate which option fits your experience on management of the impact of PRT on quality of life**	**Not at all**	**Slightly**	**Somewhat**	**A lot**	**Completely**
RT has impacted my psychological well-being	14 (26.9)	21 (40.4)	2 (3.8)	10 (19.2)	5 (9.6)
RT has impacted my social well-being	34 (65.4)	5 (9.6)	2 (3.8)	7 (13.5)	4 (7.7)
RT has impacted my physical well-being	4 (7.7)	13 (25.0)	24 (46.2)	5 (9.6)	6 (11.5)
RT has impacted my relationship(s) in terms of intimacy and communication	8 (15.4)	12 (23.1)	11 (21.2)	9 (17.3)	12 (23.1)
RT has impacted my fertility, fertility preservation and family planning	5 (9.6)	6 (11.5)	4 (7.7)	7 (13.5)	30 (57.7)
2. **For each statement below, please indicate which option fits your experience on methods of managing the impact of RT.**		**Yes**	**No**	**I am not sure**	**I prefer not to answer**
Hormone replacement therapy		19 (36.5)	33 (63.5)	0 (0.0)	0 (0.0)
Dilator use (after radiotherapy)		30 (57.7)	22 (42.3)	0 (0.0)	0 (0.0)
Pelvic floor physiotherapy		17 (32.7)	35 (67.3)	0 (0.0)	0 (0.0)
Sexual health clinic		6 (11.5)	43 (82.7)	0 (0.0)	3 (5.8)
Sexual health counseling		14 (26.9)	38 (73.1)	0 (0.0)	0 (0.0)
Martial/relationship counseling		10 (19.2)	35 (67.3)	0 (0.0)	7 (13.5)
Pain medication		19 (36.5)	28 (53.8)	0 (0.0)	5 (9.6)
Physical exercise		22 (42.3)	26 (50.0)	0 (0.0)	4 (7.7)
Fertility preservation		7 (13.5)	37 (71.2)	0 (0.0)	8 (15.4)
Dietary modification/management		11 (21.2)	35 (67.3)	0 (0.0)	6 (11.5)
Heat therapy		7 (13.5)	40 (76.9)	0 (0.0)	5 (9.6)
Psychosocial support		19 (36.5)	29 (55.8)	0 (0.0)	4 (7.7)
Skin care management		5 (9.6)	44 (84.6)	0 (0.0)	3 (5.8)
3. **For e ach statement below, please indicate which option fits your experience.**		**Not enough**	**Just enough**	**Too much**	**I have yet to receive any detail**
The information I received about symptoms and side effects of RT from my healthcare team, was it:		19 (36.5)	27 (51.9)	6 (11.5)	0 (0.0)
The information I received about sexual health counseling from my healthcare team, was it:		30 (57.7)	16 (30.8)	6 (11.5)	0 (0.0)
The education I received from my healthcare team about sexual healthcare, was it:		31 (59.6)	16 (30.8)	5 (9.6)	0 (0.0)
The education I received from my healthcare team on the sexual health resources and support available to me, was it:		38 (73.1)	12 (23.1)	2 (3.8)	0 (0.0)
4. **Which of the following described the sexual health counseling support you received?**		**Yes**	**No**	**I am not sure**	**I prefer not to answer**
Have you received sexual health counseling as part of your cancer treatment?		13 (25.0)	38 (73.1)	1(1.9)	0 (0.0)
Were you informed about the availability of sexual health counseling before or during your RT?		19 (36.5)	27 (51.9)	6 (11.5)	0 (0.0)
Are you satisfied with the quality of sexual health counseling you received?		4 (7.69)	10 (19.2)	38 (73.1)	0 (0.0)
Did sexual health counseling address your specific concerns and needs related to RT?		7 (13.5)	7 (13.5)	38 (73.1)	0 (0.0)
Did you feel comfortable discussing your sexual health concerns with your healthcare provider?		17 (32.7)	31 (59.6)	0 (0.0)	4 (7.7)
Were your healthcare providers proactive in addressing sexual health challenges you experience during or after PRT?		24 (46.1)	25 (48.1)	0 (0.0)	3 (5.8)
Did sexual health counseling help you manage any sexual health challenges you experienced during or after PRT?		5 (9.6)	9 (64.3)	38 (73.1)	0 (0.0)
Did you experience improved emotional well-being and body image because of sexual health counseling?		4 (7.69)	10 (19.2)	38 (73.1)	0 (0.0)
Were you provided with resources to support your sexual health?		20 (38.5)	27 (51.9)	5 (9.6)	0 (0.0)
Did you have access to support groups, educational resources, or peer support networks related to sexual health and PRT?		12 (23.1)	35 (67.3)	5 (9.6)	0 (0.0)

When triangulated with experiences described in the interviews, participants described how physical changes caused by pelvic RT, such as pelvic pain, stenosis and dryness led to severe, persistent pain during intercourse and loss of sexual desire, which hindered emotional intimacy and physical connection with their partners, a barrier to intimacy not adequately addressed in cancer survivorship care. One participant said,

“I’m struggling with the anticipation of pain…. Sex is very mind body and like I’m scared to have sex because I’m scared of this pain [from radiation injuries].”

Further elaborating that she had to

“figure it all out on my own”

despite receiving a vaginal dilator and information package (Table 3). Another participant shared ongoing difficulty with sexual desire, stating,

“I just didn’t have any [sexual] desire during treatment…. I still just do not have the same interest as before treatment…. after the changes my body has gone through from the radiation, I just don’t enjoy it much and barely engage in sexual activity with my partner,”

**Table 3:** Themes, Sub-themes and Participant Quotes.

Theme	Exemplification Quotes
Managing the Impact of Pelvic RT-Induced Sexual Dysfunction on Relationships and Intimacy	“I’m struggling with the anticipation of pain…. Whenever I did try like, any intercourse or whatever, it’s really painful…. Sex is very mind body and like I’m scared to have sex because I’m scared of this pain…. I was given a dilator and an information package after radiation and basically had to figure it all out on my own…. The resources felt limited…. I wish there was a therapist I could talk to long-term about the lasting pain…. work through hormonal changes with…. and manage the build-up of scar tissue that makes any sort of sexual activity too hard”. “I just didn’t have any [sexual] desire during treatment…. I still just do not have the same interest as before treatment…. after the changes my body has gone through from the radiation, I just don’t enjoy it much and barely engage in sexual activity with my partner…. more awareness on sexual health counseling would be helpful….… I didn't even know any of this stuff existed. Like, no one ever told what sexual health counselling was or that it was available.... The resources I did use I literally found myself…. maybe having access to sexual health counseling would provide me with that supportive environment I am looking for….”. “[PRT] put extra strain on our relationship…. It’s different if you have not been intimate before… now you are taking that away from our relationship…. it creates disconnect between us…. couples counseling or even some kind of partner workshop that focuses on understanding each other’s feelings…. maybe finding new ways to connect would be very helpful”. “[My partner] is very supportive, …. but it's hard to have these conversations because he just doesn't understand what it's like for me, …. not just cancer but all the parts like the radiation. He never will unless he’s in it you know…. I can tell it's frustrating for him not to be able to be intimate with me…. it’s still hard for him…. I would like to have support resources for my partner …. It’s a challenge for both of us…. Maybe there could be counseling or support groups that help him understand my experience and cope with his feelings about these changes”.
Navigating the Impact of Pelvic RT-specific SRH changes on fertility and family planning	“A lot of decisions are made for you in the process…. the treatment…. put me immediately into early menopause and made me infertile… losing that [fertility] was really tough because I wanted kids…. When you lose your ability to have children, there's a lot of grief associated with that….… I needed to grieve.... that was the one piece that I didn't get from the hospital”. “I underwent ovarian transposition prior to my treatment, thinking it would preserve my fertility….… I didn’t realize that my ovaries would be disconnected from my fallopian tubes…. I’d need IVF or that I’d have irregular periods and spotting…. we need educational resources that provide information on the potential impacts of treatment on fertility…. reproductive health and fertility options…. so, we know what to expect and can be better prepared”. “I wasn’t thinking about having a family back then [during treatment]…. I realize now…. I wasn’t just being told I have cancer…. they were also telling me I can't have kids naturally…. that's a big thing for any woman to go through…. It’s hard when actions taken then…. going forward or not with fertility preservation….… at a time when my priorities were different impact my life so much…. implementing advocacy that helps patients understand the long-term impact of these decisions…. I needed someone to really walk me through what these choices could mean for my future…. when I wasn’t in a headspace to fully see it myself”. “Before [PRT] I was told about fertility preservation…. but I couldn’t afford it…. I had to let go of that possibility…. After that initial conversation, family planning hasn’t really been brought up again…. I understand that their primary goal is to treat the tumor…. [but] starting a family is really important to me…. my overall well-being…. It's not just about surviving cancer…. it's about having a quality life afterward…. …. now that I am in follow-up, support from my healthcare team that focuses …. on goals like building a family…. would make a huge difference”.
Understanding the emotional and psychosocial toll of chronic pelvic discomfort and post-pelvic RT treatment body changes	“No one around me that understands how painful it is to even walk sometimes because of the burns…. how severe the pelvic pain can be at times…. It’s hard to go out when I’m in pain …. and discomfort…. I’d stick to staying home…. it feels isolating…. I totally think that talking to somebody who's been through it, sharing your war stories with is really helpful….… Being able to hear how they manage from day to day and adjusted to this new normal would make me feel better I think”. “You equate cancer to something that somebody who’s 60 plus has…. mentally that was a huge hurdle for me to get through…. to this add the bladder issues…. diarrhea…. even [vaginal] dryness…. all these different emotions…. all at once…. creating more depression and anxiety…. It would help to talk about these feelings openly in a conversation…. like we are right now…. to learn coping mechanisms…. through counseling or support groups or even webinars”. “I think having that one person that can almost be like a family practitioner, but within the hospital would be a huge thing….…having a point person to call to then direct you to the right place.... you don't expect them to know everything about everything, but they can point you in the right direction…. being in a dichotomy with my body…. I am figuring out how I feel in my skin and kind of who I am…. what my identity is as a young woman and someone going through all these changes after diagnosis and treatment.... I have to navigate all that as I go through this difficult journey”. “The side effects [from PRT] have made me avoid anything to do with sexual health…. I just do not see my body the same way anymore…. I don’t feel good about my body…. It adds to my anxiety… creating a resource portal with links on how to work on my sexual well-being…. what programs or objects to use…. how to use them…. maybe with testimonials from patients on what works for them”.

emphasizing the need for SRH counseling that specifically addresses radiation-related changes to sexual function and intimacy in young people AFAB (Table 3). These experiences highlight how the intersection of pelvic-specific physical changes and a lack of targeted post-treatment support amplifies the disruption to intimacy, with unique implications for AYA relationships.

Other participants noted that the pelvic RT-related sexual dysfunction further impacted their relationships, placing strain on intimate relations and barriers in communication between partners, distinct from the effects of chemotherapy and surgery. One participant noted

“[RT] put extra strain on our relationship…. It’s different if you have not been intimate before… now you are taking that away from our relationship…. it creates disconnect between us,”

referencing pelvic RT-related sexual dysfunction as the root cause. She expressed that partner-inclusive support that encompasses pelvic RT recovery, such as couples counseling, would be beneficial (Table 3). Another participant echoed similar challenges discussing these changes with their partner, stating,

“[My partner] is very supportive, …. but it's hard to have these conversations because he just doesn't understand what it's like for me, …. not just cancer but all the parts like the radiation,”

emphasizing the importance of intimacy-centered support services that help both patients and their partners understand and navigate these unique RT-related impacts (Table 3). These findings highlight the need for comprehensive, RT-specific support services for AYAs and their partners—services that recognize the distinct relational and sexual health challenges caused by pelvic RT, beyond what has been described in prior general oncology literature.

#### 3.2.2 Navigating the impact of pelvic RT-specific SRH changes on fertility and family planning

Most respondents (87%, n = 45) experienced fertility issues and (85%, n = 44) faced emotional distress after RT (Table 2), with 90% (n = 47) describing that RT impacted their fertility and family planning. Participants did not view infertility as a single outcome but described how pelvic RT caused unexpected changes to their reproductive health, including anatomical, hormonal, and functional effects that differed from those seen with systemic treatments. Many (71%, n = 37) did not undergo fertility preservation, (64%, n = 33) did not use hormone replacement therapy, nor attended a SRH clinic post-treatment (83%, n = 43). Many respondents (60%, n = 31) reported feeling uncomfortable discussing their SRH concerns with their healthcare provider and 52% (n = 27) felt that they were not provided with resources to support their SRH. Interviews confirmed these RT-specific SRH changes created new and evolving challenges in fertility and family planning that extended beyond the immediate post-treatment period.

For example, one participant expressed the emotional toll of unanticipated infertility, sharing how

“radiation treatment put me immediately into early menopause and made me infertile,”

making the loss especially difficult given their desire for children. They articulated a desire for grief counseling sharing

“When you lose your ability to have children, there's a lot of grief associated with that….… that was the one piece that I didn't get from the hospital” (Table 3).

Other participants also expressed similar changes in reproductive function due to pelvic RT and the need for resources specific to their SRH, including unanticipated reproductive changes despite undergoing proactive steps like ovarian transposition. They shared their frustration, explaining that they

“didn’t realize that my ovaries would be disconnected from my fallopian tubes”

and that IVF would be necessary. This highlights the need for education specifically about the anatomical consequences of pelvic RT on reproductive structures, which participants felt was inadequately addressed in standard fertility counseling. They emphasized the need for educational resources to better prepare patients for potential reproductive health impacts, saying we need “information on the potential impacts of treatment on fertility…. so we know what to expect” (Table 3).

For these participants, they desired more support and understanding regarding the long-term, evolving impacts of pelvic RT on reproductive health and the complexity of future family-building options. One participant explains that during treatment

“I wasn’t thinking about having a family back then [during treatment]…. I realize now…. they were also telling me I can't have kids naturally”.

They highlighted the need for greater advocacy and decision-making support, adding

“I needed someone to really walk me through what these choices could mean for my future” (Table 3).

Another participant also requested tailored supports for fertility changes relating to RT sharing and also noted that after the initial discussion,

“family planning hasn’t really been brought up again… and it's not just about surviving cancer…. it's about having a quality life afterward” (Table 3).

Collectively, these findings highlight a novel gap in survivorship care—the need for longitudinal, RT-specific education and holistic, ongoing support that addresses the delayed realization of fertility loss, its emotional impact, and broader goals related to family planning, reproductive health, and quality of life after cancer.

#### 3.2.3 Understanding the emotional and psychosocial toll of chronic pelvic discomfort and post-pelvic RT treatment body changes

Among survey respondents, 92% (n = 48) reported anxiety, 89% (n = 46) faced depression, and 81% (n = 42) expressed body image concerns because of SRH challenges they currently experience. Additionally, 73% (n = 38) experienced persistent pelvic pain, 75% (n = 39) reported vaginal discomfort, 67% (n = 35) experienced vaginal dryness and 40% (n = 21) suffered vaginal stenosis (Table 2), highlighting the enduring and uniquely localized impacts of pelvic RT on sexual and pelvic health. RT was reported to impact psychological wellbeing of 73% (n = 38), while 35% (n = 18) experienced impacts on their social wellbeing and 92% (n = 48) felt that RT had impacted their physical well-being due to ongoing discomfort and physical limitations. Despite these significant impacts, 67% (n = 35) had not tried pelvic floor physiotherapy, and 50% (n = 29) had not engaged in physical exercise to manage the impact of RT, 37% (n = 19) felt that the information they received about pelvic symptoms and side effects of RT was not adequate. Moreover, 60% (n = 31) indicated that the education they received from their healthcare team about SRH care did not address the long-term physical sequelae unique to pelvic RT, including chronic pain, bowel and bladder dysfunction, and sexual side effects**.** Although 48% (n = 25) of survey responses felt that their healthcare providers were proactive in addressing SRH challenges they experienced during or after treatment, most (67%, n = 35) did not have access to RT-specific support groups, educational resources or peer support networks to help navigate these chronic, post-treatment impacts on their daily lives. Interviews expanded on these findings, highlighting that the physical and functional pelvic changes following RT were deeply intertwined with participants’ emotional, psychological, and social well-being in ways not previously captured in existing literature.

This was true for one participant who noted feeling alone in their experience of chronic pelvic discomfort, saying no one understood

“how painful it is to even walk sometimes”

or how severe the discomfort could be during routine activities (Table 3). The pain limited their ability to engage socially, adding,

“It’s hard to go out when I’m in pain…. I’d stick to staying home…. it feels isolating”.

They emphasized the absence of RT-specific peer support, sharing that

“talking to somebody who’s been through it”

would help normalize these experiences and facilitate adjustment to this

“new normal”.

Another participant encountered mental health challenges due to RT side effects, explaining that they never expected to face such challenges at their age, including bladder issues, diarrhea, and vaginal dryness (Table 3).

They emphasized the importance of open discussions, counseling, and peer groups, sharing,

“It would help to talk about these feelings openly…. to learn coping mechanisms…. through counseling or support groups or even webinars”,

highlighting the importance of accessible psychosocial supports specific to pelvic RT sequelae.

These participants, as was true for others, spoke of needing tailored support and information to navigate the emotional challenges of grappling with identity and body image changes following the physical changes caused by pelvic RT treatment. One participant desired having a patient navigator to help navigate them post-RT resources, articulating it would help to have.

“a point person to call”

who could direct them to tailored services (Table 3). They spoke of the internal conflict of adjusting to their post-treatment body and identity shift; another saying they avoided sex entirely.

“I am figuring out how I feel in my skin and kind of who I am…. what my identity is as a young woman…. after diagnosis and treatment”.

“I just do not see my body the same way anymore…. I don’t feel good about my body…. It adds to my anxiety” (Table 3).

They advocated for resources specifically focused on the chronic physical and sexual changes following pelvic RT, including practical guidance, educational tools, and patient testimonials to help normalize these experiences.

While many participants reported challenges, some highlighted positive experiences that supported their SRH post-RT. Participants appreciated when healthcare providers-initiated discussions about SRH and in being given clear guidance on managing changes.

“My oncologist actually brought it up first, which made me feel like it was okay to talk about.”

“I was helpful when my nurse explained how to use dilators properly…. it made the process feel less overwhelming.”

These experiences emphasize the impact of proactive provider communication, clear patient education, and rehabilitative care in supporting SRH after RT.

## 4 Discussion

This is the first known study to examine in-depth the SRH experiences, challenges, and concerns of AYA gynecologic oncology patients AFAB, specifically those with cervical cancer, following pelvic RT, with a focus on patient-identified care gaps and proposed solutions. We found that AYAs AFAB face significant, often under-addressed changes in SRH, including intimacy, fertility, and psychosocial well-being. By centering patient narratives and triangulating survey and interview data, our findings reveal RT-specific impacts often missed in broader oncology literature and offer actionable recommendations from the patient perspective. Because most participants received multimodality therapy, our findings reflect patient-attributed and RT-anchored experiences rather than isolated causal effects of RT alone.

This study is novel in its focus on AYA AFAB gynecologic oncology patients post-pelvic RT, a population underrepresented in research despite unique SRH challenges related to anatomy, hormones, and psychosocial development. Unlike prior studies that broadly address cancer-related sexual dysfunction, most of which focus on older adult populations or mixed-age cohorts, our findings highlight how similar symptoms (e.g., pelvic pain, vaginal dryness, dyspareunia) carry different meaning and consequences for AYAs, who are more likely to be navigating dating, early partnerships, family planning, and identity formation. W**e** directly link SRH disruptions to pelvic RT and capture the longitudinal ways they affect identity, intimacy, and family-building. Whereas studies in older populations often emphasize symptom burden and functional outcomes, our participants emphasized disruption to life trajectory, relationships, and future planning as equally central harms. While these experiences occur in the context of combined treatment, participants consistently framed and interpreted many of these disruptions in relation to their RT exposure. Participants also proposed practical, RT-specific solutions, like partner-inclusive counseling, peer-led education, and survivorship navigation that have not been emphasized in prior literature.

Participants described substantial challenges maintaining intimacy and communication due to sexual dysfunction such as pain during intercourse, decreased libido, and fear of sexual activity (Jensen and Froeding, 2015, Pup et al., 2019). These difficulties strained relationships and caused emotional distress, worsened by limited access to tailored SRH counseling or partner-focused support (Dizon et al., 2024, Bober and Varela, 2012). While AYAs undergoing other treatments face similar struggles (Cherven et al., 2024), our participants directly attributed relational issues to pelvic RT. We acknowledge that these symptoms may be influenced by surgery and/or systemic therapy; however, the consistent patient attribution to RT highlights how radiation-related pelvic changes are perceived as a central driver of these difficulties. Although dyspareunia and pelvic pain are also reported in older survivors, AYAs in this study more often described these symptoms as barriers to relationship formation, intimacy development, and sexual confidence, rather than solely as treatment side effects to be managed. They highlighted a lack of RT-specific rehabilitation and couples-based services as a key care gap. This highlights the need for interventions like couples counseling, partner workshops, and SRH education that reflect the distinct anatomical and functional changes of pelvic RT. Ongoing, age-appropriate support that includes partners is essential to improving intimacy and relationship quality.

Consistent with previous work, participants reported profound emotional distress due to premature ovarian failure and infertility including grief, regret, and a lack of preparedness (Wo and Viswanathan, 2009, Reiser et al., 2024, Logan and Anazodo, 2019). Like earlier studies, our findings emphasize persistent gaps in fertility preservation discussions and educational resources (Park et al., 2009, Romao and Lorenzo, 2017). However, what sets this study apart is its focus on the multifactorial RT-related fertility distress including anatomical disruption, hormonal dysfunction, and delayed regret. Although fertility outcomes in this population are shaped by multiple treatments, participants frequently situated their reproductive concerns within the context of pelvic RT and its anticipated or experienced effects. Compared with older populations, for whom fertility may be less immediately relevant, AYAs described infertility as a threat to future identity, life plans, and perceived normalcy, intensifying its psychological impact. Participants emphasized a need for better education on reproductive impacts, fertility preservation, and long-term options, along with emotional support such as grief counseling and decision-making advocacy. These patient-identified solution including RT-specific fertility counseling and survivorship tools, which are novel contributions not captured in earlier studies. Rather than focusing solely on fertility outcomes, this study reveals the ongoing psychological impact of infertility on AYA survivors.

Participants also described high levels of psychosocial distress, including anxiety, depression, body image issues, and decreased self-esteem, all linked to chronic pelvic discomfort and functional limitations (Amarnath, 2023, Adams et al., 2014).These physical symptoms were deeply intertwined with emotional challenges and social isolation (Akeflo et al., 2022). While prior studies note psychological distress in cancer survivors (Abdelhadi, 2023), our findings uniquely demonstrate how pelvic RT-related dysfunction intersects with identity, social roles, and mental health in AYAs. We recognize that these psychosocial outcomes emerge within the broader context of multimodality cancer treatment; however, participants repeatedly identified RT-related pelvic symptoms as a key contributor to their ongoing distress. In contrast to older survivors, who may frame these symptoms in the context of aging or comorbidity, AYAs often experienced them as premature, stigmatizing, and disruptive to developmental milestones such as career building, partnership formation, and family planning. This highlights the urgent need for targeted psychosocial interventions, peer networks, and educational tools. Participants specifically requested practical guidance for symptom management, relationship communication, and navigating identity after treatment. Critically, they identified patient navigators, RT-specific resources, and peer-led supports as essential tools for managing survivorship. This underscores the potential value of structured support groups and navigation programs that address the physical, emotional, and sexual challenges AYAs face post-RT.

This study has limitations. Conducted at a single academic cancer center, findings may not be generalizable to other settings (Stalmeijer et al., 2024). Our cross-sectional design limits insight into how SRH concerns evolve over time (Germeni et al., 2015). Although thematic saturation was achieved, we may not have captured the full diversity of AYA experiences, especially among culturally or socioeconomically diverse groups (Giuliano et al., 2010). Selection bias is possible, as participants may have been more likely to engage due to strong views or significant toxicity (Walsh et al., 2012), and the low survey response rate (52/73, 70.3%). Most were cisgender and heterosexual, underscoring the need for future studies inclusive of sexual and gender-diverse AYAs. Additionally, the survey and interview tools were not pilot-tested or validated, which may affect the precision of findings. There was no patient involvement in tool development, which may limit alignment with patient priorities or language. Importantly, because most participants received surgery and/or systemic therapy in addition to RT, the effects of RT cannot be fully isolated; thus, our findings should be interpreted as patient-attributed, RT-anchored experiences within multimodality care rather than definitive causal effects of RT alone.

In conclusion, this study is the first to comprehensively document the sexual, reproductive, and psychosocial impacts of pelvic RT on AYA AFAB survivors. Through mixed-methods analysis, we identified key care gaps and patient-desired solutions, including partner-inclusive care, RT-specific education, and survivorship support. These findings reflect how patients experience and interpret RT-related impacts within the broader context of combined cancer treatment**.** By highlighting how similar symptoms carry different developmental, relational, and existential consequences for AYAs compared with older adults, this study emphasizes the need for age-specific, life-stage–informed models of survivorship care. Our findings emphasize the need for tailored interventions that address not only physical and sexual dysfunction, but also the identity, mental health, and long-term reproductive goals of this population. Future efforts should prioritize development of RT-specific resources—such as multimedia tools, peer support programs, and patient navigators—to ensure holistic, patient-centered care for AYA AFAB survivors of pelvic RT.

## 7 Data availability statement

The data presented in this study are available on request from the corresponding author.

## CRediT authorship contribution statement

**Kaviya Devaraja:** Writing – review & editing, Writing – original draft, Validation, Project administration, Methodology, Investigation, Formal analysis, Data curation, Conceptualization. **Anjali Sachdeva:** Writing – review & editing, Writing – original draft, Validation, Investigation, Formal analysis. **Karina Gandhi:** Writing – review & editing, Writing – original draft, Validation, Investigation, Formal analysis. **Abha A. Gupta:** Writing – review & editing, Writing – original draft, Validation, Supervision, Project administration, Methodology, Investigation, Data curation, Conceptualization. **Jennifer Croke:** Writing – review & editing, Writing – original draft, Validation, Supervision, Project administration, Methodology, Investigation, Data curation, Conceptualization.

## Ethics Approval

This study was performed in line with the principles of the Declaration of Helsinki. Ethics approval was obtained from the University Health Network (UHN) Research Ethics Board (REB) on February 26, 2024 (CAPCR # 22-6012).

**Consent to Participate**

Written, informed consent was obtained from all individual participants included in the study.

**Consent to Publish**

The participants consented to the publishing of their data in the journal.

## Funding

This work was supported in part by the Adolescent and Young Adult Program, Princess Margaret Cancer Foundation.

## Declaration of Competing Interest

The authors declare that they have no known competing financial interests or personal relationships that could have appeared to influence the work reported in this paper.

## Glossary

Adolescent and Young Adult (AYA): Individuals aged 15–39 years at the time of cancer diagnosis, representing a distinct group with unique developmental, psychosocial, and healthcare needs.
Assigned Female at Birth (AFAB): A term used to describe individuals designated as female when born, regardless of current gender identity.
Brachytherapy: A type of internal radiotherapy where a radioactive source is placed directly inside or next to the treatment area.
External Beam Radiotherapy (EBRT): A radiation treatment technique where high-energy beams are directed from outside the body to target cancer cells within the pelvis.
Fertility Preservation: Medical interventions performed before or during cancer treatment to maintain the ability to have biological children in the future (e.g., egg or embryo freezing, ovarian transposition).
Pelvic Radiotherapy (Pelvic RT): Radiation treatment delivered to the pelvic region to manage cancers involving pelvic organs, such as cervical, uterine, or rectal cancers.
Premature Ovarian Failure (POF): Loss of normal ovarian function before age 40, often resulting from pelvic RT, leading to infertility and early menopause.
Sexual and Reproductive Health (SRH): A state of physical, emotional, mental, and social well-being related to sexuality and reproduction, encompassing sexual function, fertility, and autonomy in reproductive decisions.
Sexual Dysfunction: Persistent difficulties in sexual desire, arousal, orgasm, or pain during sexual activity that cause distress or impair relationships.
Vaginal Stenosis: Narrowing and shortening of the vaginal canal due to fibrosis and scarring, commonly resulting from pelvic radiotherapy.
Vaginal Dilator: A medical device used to prevent or reduce vaginal stenosis after pelvic radiotherapy by maintaining vaginal elasticity and patency.
Mixed-Methods Study: A research design that integrates both quantitative (numerical) and qualitative (narrative) data to provide a comprehensive understanding of a phenomenon.
Thematic Analysis: A qualitative analytic method used to identify, analyze, and interpret patterns or themes within interview data.
Triangulation: A process in mixed-methods research that combines multiple data sources or methods to enhance the validity and depth of findings.
Survivorship Care: A phase of cancer care focusing on the health and life of a person post-treatment, addressing ongoing physical, psychosocial, and reproductive concerns.
Partner-Inclusive Counseling: A therapeutic approach that involves both the patient and their partner to address relational and sexual challenges after cancer treatment.
Pelvic Floor Physiotherapy: A rehabilitative therapy aimed at improving the function and strength of pelvic muscles affected by radiotherapy or surgery.
Hormone Replacement Therapy (HRT): Treatment involving hormones (e.g., estrogen) to alleviate symptoms associated with premature menopause or ovarian failure.
Body Image Disturbance: Negative perception or dissatisfaction with one’s body, often resulting from treatment-induced physical or sexual changes.
Psychosocial Distress: Emotional suffering related to the psychological and social effects of illness, including anxiety, depression, and social isolation.
