# Supplementary material for: Radiation-specific sexual and reproductive health challenges in adolescent and young adults assigned female at birth: a mixed-methods study of unmet needs post pelvic radiotherapy
Source: Gynecol Oncol Rep. 2026 Feb 22;64:102051. doi: 10.1016/j.gore.2026.102051 (PMC12955145; doi:10.1016/j.gore.2026.102051)
Supplement: Supplementary Data 1 [file mmc1.docx]

Supplementary Tables

Supplemental Table 1: Online survey: questions

| Part 1: Demographics |  | |
| --- | --- | --- |
| Question | Response Choices | |
| 1. Date of birth | Month/year  I prefer not to answer | |
| 1. Where were you born? | Canada  Other, please specify  I prefer not to answer | |
| 1. What is your racial or ethnic origin? (select all that apply) | Arab First Nations/Indigenous  Latin American Middle eastern  Canadian Caucasian (White)  North American South American  American Australian  Oceanian New Zealander  Northern European Eastern European  Western European Southern European  Southeast European Latin American  Central African West African  North African East African  African American African Canadian  African Caribbean Caribbean  South Asian Southeast Asian  West Asian Central Asian  East Asian Eurasian  Other, please specify I prefer not to answer | |
| 1. What languages do you speak? | English French Spanish  Punjabi Portuguese Hindi  Chinese (Mandarin and Cantonese) Tagalog  Urdu Italian Korean  Arabic Russian Tamil  Farsi German Bengali  Indigenous Languages Vietnamese  Other, please specify I prefer not to answer | |
| 1. What is your gender? | Man Non-binary  Woman Two-spirit  Transgender woman Other, please specify  Transgender man I prefer not to answer | |
| 1. What is your sexual orientation? | Heterosexual Gay Lesbian  Bisexual Pansexual Asexual  Queer  Other, please specify I prefer not to answer | |
| 1. Are you sexually active? | Yes No  Other, please specify I prefer not to answer | |
| 1. What is your relationship status? | Partnered Not partnered  Other, please specify I prefer not to answer | |
| 1. Does your partner have sexual challenges related to your sexual dysfunction? | Yes No  Other, please specify I prefer not to answer | |
| 1. Please select the option that best describes where you live | Rural (population of less than 50,000)  Small town (population from 50,000 to 250,000)  Large city (population from 250,000 to 1 million)  Metropolitan Centre (population of 1 million or more)  Other, please specify  I prefer not to answer | |
| 1. What is your highest degree or level of school completed | No schooling Graduate/professional degree  Highschool diploma Other, please specify  University/college degree I prefer not to answer | |
| 1. What is your main activity? | Working Student  Homemaker On disability  Other, please specify I prefer not to answer | |
| 1. Which of the following best describes where you are with your care/treatments? | You have yet to start cancer treatment  You are currently in active treatment  You have completed treatment and in long-term follow-up  You have been discharged from your cancer centre  You are unsure  Other, please specify  I prefer not to answer | |
| 1. Please select all the treatments that you have received | Chemotherapy Hormone therapy  Radiation Targeted therapy  Surgery Other, please specify  I prefer not to answer | |
| 1. What other health conditions do you have? | Diabetes High cholesterol  Hypertension Thyroid  Obesity Cardiovascular disease  Another cancer diagnosis Mental illness  Eating disorders Drug & Alcohol abuse  Autoimmune disease STI/STD  Other, please specify I prefer not to say | |
| 1. What is your employment status | Full-time Other, please specify  Part-time I prefer not to answer  On disability | |
| 1. What is your yearly household income before taxes? | Less than $30,000 $215,000-$299,999  $30,000-$60,999 Above $300,000  $61,000-$90,000 Other, please specify  $90,000-$150,000 I prefer not to answer  $151,000-$214,999 | |
| Part 2: AYA Sexual Health Assessment |  | |
| 1. What changes to sexual health do you experience NOW? Please rank the following 1-5. | 1. Vaginal dryness 2. Vaginal stenosis 3. Vaginal discomfort 4. Pain during sex 5. Loss of libido or sexual desire 6. Loss of enjoyment in sexual activity 7. Loss of engagement in sexual activity 8. Changes in orgasms or intensity of orgasms 9. Anxiety 10. Depression 11. Emotional distress 12. Body image concerns 13. Loss of intimacy 14. Deterioration of relationship communication 15. Changes in self-esteem 16. Fatigue 17. Fertility issues 18. Pelvic pain 19. Bowel Incontinence 20. Lymphedema 21. Diarrhea 22. Cramping | Frequency of symptom experience     - Not at all - Rarely - Sometimes/ Occasionally - Frequently/ Often - Always - Other, please specify - I prefer not to answer |
| 1. Are there other symptoms that you experience with PRT? If yes, please specify. | No  Yes, please specify  I prefer not to answer | |
| 1. For each statement below, please indicate which option fits your experience on management of the impact of PRT on quality of life | 1. PRT has impacted my psychosocial well-being 2. PRT has impacted my social well-being 3. PRT has impacted my physical well-being 4. PRT has impacted my relationship(s) in terms of intimacy and communication 5. PRT has impacted my fertility, fertility preservation and family planning | - Not at all - Slightly - Somewhat - A lot - Completely - Other, please specify - I prefer not to answer |
| 1. For each statement below, please indicate which option fits your experience on methods of managing the impact of PRT. | 1. Hormone replacement therapy 2. Dilator use (after radiotherapy) 3. Pelvic floor physiotherapy 4. Sexual health clinic 5. Sexual health counseling 6. Martial/relationship counseling 7. Pain medication 8. Physical exercise 9. Fertility preservation 10. Dietary modifications/management 11. Heat therapy 12. Psychosocial support 13. Skin care management | - Yes - No - Other, please specify - I prefer not to answer |
| Part 3: AYA Care at Princess Margaret Cancer Centre |  |  |
| 1. For each statement below, please indicate which option best fits your experience. | 1. The information I received about symptoms and side effects of PRT from my healthcare team was it: 2. The information I received about sexual health counseling from my healthcare team, was it: 3. The education I received from my healthcare team about sexual healthcare, was it: 4. The education I received from my healthcare team on the sexual health resources and support available to me, was it: | - Not enough - Just enough - Too much - I have yet to receive any detail - Other, please specify - I prefer not to answer |
| 1. Which of the following described the sexual health counseling support you received? | 1. Have you received sexual health counseling as part of your cancer treatment? 2. Were you informed about the availability of sexual health counseling before or during your PRT? 3. Are you satisfied with the quality of sexual health counseling you received? 4. Did sexual health counseling address your specific concerns and needs related to PRT? 5. Did you feel comfortable discussing your sexual health concerns with your healthcare provider? 6. Were your healthcare providers proactive in addressing sexual health challenges you experience during or after PRT? 7. Did sexual health counseling help you manage any sexual health challenges you experienced during or after PRT? 8. Did you experience improved emotional well-being and body image because of sexual health counseling? 9. Were you provided with resources to support your sexual health? 10. Did you have access to support groups, educational resources, or peer support networks related to sexual health and PRT? | - Yes - No - Other, please specify - I prefer not to answer |

Supplemental Table 2: Interview guide script: questions and probes.

| Part 1 – PRT Experience    Question     - What was it like to receive PRT?     Probes:   1. Do you face any challenges to your sexual health? If so, what are the challenges you face? 2. Do you experience any changes to your sexual health? If so, what are the changes you experience? 3. Do you face any negative impact on your day to day and overall quality of life? If so, what negative impacts do you face? How does this impact your sexual health? 4. Do you experience any symptoms or side effects that impact your sexual health? If so, what are the symptoms or side-effects you experience? |
| --- |
| Part 2 – PRT Impact on Sexual Health    Question     - What are the impacts that PRT has on your sexual health?     Probes:   1. Does it affect your sexual desire and function? Can you expand? 2. Does it cause any pain, discomfort or vaginal changes? If so, can you explain? 3. What is the psychological and emotional impact treatment has had on your sexual health? 4. Does it affect your fertility and family planning? If so, is this of importance to you? 5. Did this treatment impact your relationships? If so, how? Were you able to discuss your concerns with your partner? Did you HCP address the potential impact of treatment on relationships? 6. Are you asked about sexual health during follow up appointments? Discuss.... |
| Part 3 – Sexual Health Education    Question     - What is your level of understanding of the effects of PRT on sexual health outcomes?     Probes:   1. Is there anything you wish you knew more about? Can you expand 2. What do you know regarding its impact, symptoms, side effects, and effect on quality of life? 3. Have you received information or education from your healthcare providers about the sexual health changes associated with PRT? If so, how well-informed do you feel about the sexual health changes and challenges that may arise from this treatment? 4. Do you feel that your questions, concerns, and uncertainties about the effects of PRT on sexual health were adequately addressed? 5. What are your opinions on education and awareness of sexual healthcare among adolescent young adult community? 6. What changes would you like to see in the sexual health education, awareness, and advocacy for patients like yourself? 7. When would you like to receive this information and in what delivery format? |
| Part 4 – Sexual Health Care at PM  Question     - What are your personal experiences as a Princess Margaret Cancer Center patient?     Probes:   1. What are your thoughts and opinions regarding research about sexual health of adolescent and young adult patients such as yourself at Princess Margaret Cancer Center? 2. What are your thoughts and opinions about the amount of research and resources directed towards this community? 3. What gaps exist? 4. What changes would you like to see? 5. What do you think is done well at Princess Margaret in terms of, treatment, resources made available, and research conducted on sexual health of adolescent and young adult patients such as yourself receiving PRT? 6. What gaps exist? 7. What changes would you like to see? 8. What are your thoughts and opinions about sexual health counseling provided to adolescent and young adult patients such as yourself at Princess Margaret Cancer Center? 9. What support was provided? 10. What gaps exist? 11. What changes would you like to see? 12. What are your thoughts and opinions on support and communication with healthcare providers at Princess Margaret regarding your sexual health concerns? 13. Is there open communication? Can you elaborate? 14. Are your treatment goals addressed and prioritized? If so, can you expand on this? 15. What gaps exist? 16. What changes would you like to see? 17. Are there any other gaps that exist? Can you speak about any gaps that exist? 18. Are there any changes you would like to see? What changes would you like to see? |
| Part 5 – Sexual Health Specific Resources and Support  Question   - What resources, and/or support services are you accessing Princess Margaret Cancer Center?   Probes:   1. What are your opinions on the current services made available? 2. Are/were you provided with adequate guidance, resources, and support to address sexual health concerns during and/or after treatment? Can you elaborate on these experiences? 3. Are there any gaps that exist? If yes, what are these gaps? 4. Are there any changes you would like to see? If yes, what changes would you like to see? Why? 5. Are there any types of services/support you would like to see? Can you elaborate on what these services might look like? 6. If any, what tools do you have access to at Princess Margaret Cancer Center? 7. Do these tools educate you on sexual dysfunction diagnosis, treatment options, statistics, research, and resources available to adolescent and young adult patients at Princess Margaret Cancer Center? 8. Are there tools you would you like access to in order to improve your overall health care experience, course of disease, quality of life? 9. If yes, what would these tools include? 10. Would you like the tool to allow you to connect to other patients? Why or why not? |
| Final Comments     - Is there anything else that you would like to add? Is there any important issue that we have not talked about and that you would like to share with me? |
